# Effect of four direct pulp capping agents on human dental pulp tissue

**DOI:** 10.6026/973206300200785

**Published:** 2024-07-31

**Authors:** Prerna Goel, Navneet Kukreja, Sushruth Nayak, Swati Chhabra, Surinder Sachdeva, Urvashi Sukhija

**Affiliations:** 1 Department of Conservative and Endodontics, M.M College of Dental Sciences and Research, Mm (Deemed to be University), Mullana, Ambala, India; 2 Department of Oral & Maxillofacial Pathology, Mm College of Dental Sciences and Research, Maharishi Markandeshwar (Deemed to be University), Mullana (Ambala), Haryana, India; 3 Department of Periodontics, Mm College of Dental Sciences and Research, Maharishi Markandeshwar (Deemedto be University), Mullana (Ambala), Haryana, India; 4 Department of Prosthodontics, Mm College of Dental Sciences and Research, Maharishi Markandeshwar (Deemed to be University), Mullana (Ambala), Haryana, India

**Keywords:** Direct pulp capping agents, human dental pulp tissue

## Abstract

Direct pulp capping (DPC) is a vital pulp therapy, wherein accidental or carious pulp exposures can be capped with various materials to induce reparative dentine formation. One of the major factors that determine the success of direct pulp capping procedures is the material used. Therefore, it is of interest to evaluate and compare the efficacy of a new material, Tristrontium aluminate in comparison with the widely used materials such as ProRoot MTA, Biodentine & TheraCal LC. Hence, 40 premolars scheduled for extraction for orthodontic treatment, were equally divided into 4 groups and iatrogenic DPC was done using one of the study materials. The premolars were then extracted after 90 days & assessed for the formation of Dentine Bridge and pulpal response elicited by the materials using CBCT and histopathological analysis. All the materials tested in the study were successful in inducing dentine bridge formation & maintaining pulp vitality. Thus, the novelty of Tristrontium aluminate for potential applications in future is eminent.

## Background:

Preserving pulp vitality is crucial for maintaining tooth structural integrity and physiological function as it has ability to produce secondary dentin, peritubular dentin & reparative dentin in response to mechanical, thermal, chemical, or bacterial stimuli. [1] Direct pulp capping is a conservative approach to treatment that is very often practiced for saving tooth vitality with iatrogenic pulp exposure. [2] Direct pulp capping when done properly by providing a proper biological seal, gives pulp a chance to exert its healing potential by cellular reorganization and dentinal bridge formation. [1] The ideal properties for a pulp capping material are biocompatibility, radio-opacity providing a biological seal, prevention of bacterial leakage, stimulation of dentin bridge formation, maintaining pulp vitality, and release of fluoride to prevent secondary caries. [3] First introduced in 1920, by Hermann, calcium hydroxide has been looked at as the gold standard for pulp capping. [4, 5] Recently, other biomimetic materials such as ProRoot MTA, MTA Angelus, RetroMTA, TheraCal LC, Biodentine, etc., are being used. [4] MTA has remineralizing abilities while maintaining pulp vitality. Biodentine was originally designed to substitute dentin. It is highly biocompatible and provides a superior biological seal. [6,7] TheraCal LC, is a light cured resin reinforced calcium silicate-based material, that has remineralizing abilities owing to its calcium and hydroxide ion-releasing potential. [6] There has been increased interest in strontium (Sr)-based bioactive cements as a new alternative Tristrontium aluminate is a material of choice as strontium ions behave like calcium ions and can be assimilated in hydroxyapatite crystals thus aiding in remineralization and making the tooth more acid resistant. [8] Therefore, it is of interest to evaluate the efficacy of four different direct pulp capping agents on premolars using CBCT and histopathological evaluation. The null hypothesis was that among pulp-capping agents, CBCT and histopathological evaluation have no significant difference.

## Materials and Methodology:

Approval of this prospective randomized clinical trial was taken by the Institutional Ethical Committee (vide letter no. IEC: 2264) and was registered with www.clinicaltrial.gov (ClinicalTrial.gov Identifier - NCT06435065). Informed consent was obtained from the patients who fulfilled the inclusion criteria. The sample size was calculated at 40, using G power Software (version 3.1.2) with an effect size of 0.601, 5% level of precision, 95% confidence level, and 80% power of the study. The patients were divided into 4 groups to undergo direct pulp capping procedures utilizing one of the study materials:

Group I: (n=10) Biodentine,

Group II: (n=10) MTA,

Group III: (n=10) TheraCal LC,

Group IV: (n=10) Tristrontium Aluminate.

## Inclusion & Exclusion criteria:

Healthy patients with no systemic conditions, within the age group of 15-25, undergoing orthodontic treatment and have caries-free (no signs and symptoms of pulpal inflammation or periapical pathology), undamaged, fully erupted, mature maxillary and mandibular premolars, planned for extraction as a part of orthodontic treatment. Premolars showing signs and symptoms of irreversible pulpitis, or showing signs of caries, periapical pathology, internal/external root resorption, furcal radiolucency/ inter-radicular bone destruction and/or calcifications in the pulp chamber or canals on radiographic examination and medically compromised and pregnant patients were excluded. Radiographic and clinical evaluation of the premolars was carried out including electric pulp testing. Standardized class I cavities (3.0±0.2mm in occlusal depth, 4.0±0.5mm mesiodistally & 3.0±0.2mm buccolingually), were prepared and pulp exposure measuring approximately 0.5mm was done. Hemostasis was achieved by placing normal saline-soaked sterile cotton pellets in cavity for 60 secs. Direct pulp capping was performed on the exposed pulp, according to the different groups:

## Thera Cal LC:

Material was dispensed from syringe and light cured for 20 seconds.

## White MTA:

The powder was mixed with normal saline in a ratio of 3:1 and applied over the exposure site using MTA carrier, followed by temporary restoration. Final restorative procedure was carried out on next day as per the study protocol.

## Biodentine:

The material was applied to the exposure site and was allowed to set for 9-12 minutes.

## Tristrontium aluminate:

The powder was mixed with distilled water in a ratio of 0.6 to obtain a paste-like consistency and was applied to the exposure site, as per S Adel *et al.*'s protocol.

## Restorative procedure:

The teeth were restored with RMGIC base & Filtek P60 posterior light cure composite material. Following the procedure, the patients were recalled on day 1 and after 1 week. Teeth with signs and symptoms of irreversible pulpitis were excluded from the study.

## Specimen preparation and examination:

The pulp-capped teeth were extracted a traumatically after 90 days and after cleaning mounted in wax blocks and evaluated using CBCT and histological examination and were viewed under a microscope For degree of pulpal inflammation and CBCT scoring was done as adapted from Muruganandhan *et al.* [9].

## Statistical analysis:

Data was analyzed using Statistical Package for Social Sciences (SPSS). Categorical outcome variables were analyzed by Chi-square test. Pairwise was done using Mann-Whitney U test. (p<0.05)

## Results:

On comparing the data obtained by CBCT, the decreasing order of efficacy was: Group I (Biodentine)>Group II (MTA) ≥Group IV (Tristrontium aluminate)> Group III (TheraCal LC). On comparing the data obtained by histopathological analysis, regarding the quality of the dentine bridge formed, the decreasing order of efficacy was: Group I (Biodentine) = Group IV (Tristrontium Aluminate) = Group II (MTA) > Group III (TheraCal LC). The difference in the pulpal response exerted by the materials in the study was statistically insignificant. On comparing the data obtained regarding the pulpal response by histopathological analysis, the decreasing order of efficacy regarding inflammation was: Group III (TheraCal LC)>Group I (Biodentine) = Group IV (Tristrontium Aluminate) ≥Group II (MTA) (Table 1).

## Discussion:

Direct pulp capping is a treatment protocol wherein a dental material is applied directly onto the exposed pulpal tissue, to induce formation of reparative dentine and maintain the vitality of the tooth. The success of direct pulp capping procedures majorly depends on the material used [10, 11-12]. Tristrontium aluminate has highest strontium content and is an alkaline earth element which can get incorporated into tooth hydroxyapatite, enhancing acid resistance and facilitating remineralization. [13] S Adel *et al.*, found that The S3A extract did not hinder the proliferation of MDPs and demonstrated the attachment of MDPs on its surface [8]. In the present study, CBCT and histopathological assessment were employed to gauge the success of direct pulp capping procedure. Histopathological examination is considered the gold standard for evaluation of the pulpal response to any test material, and CBCT provide 3-dimensional approach & eliminate the superimposition of surrounding tissues over one another, thereby helping in a more precise assessment of the tertiary dentin formation [2]. Nowicka *et al.* and Jayanandan *et al.* in their studies evaluated dentin bridge formation utilizing CBCT imaging and then compared the results with histopathologic analysis [14]. As per the data from CBCT, complete dentine bridge was high in Biodentine and least in TheraCal LC. In histological evaluation, equal number of cases with complete bridge resembling dentine-like mass with Biodentine, MTA, and Tristrontium aluminate, however, TheraCal LC showed the least number of cases. The superior performance of Biodentine over MTA can be attributed to higher Calcium release of Biodentine (18.0-95.3ppm) and superior ability to enhance the viability, adhesion, and migration of human dental pulp stem cells (DPSCs) [15,16]. The study's results align with findings from Dominguez *et al.* and Peskersoy *et al.*, who concluded that TheraCal LC is effective for indirect pulp capping [17,18]. In contrast, Kim *et al.* found greater mineralized nodule formation with TheraCal LC and ProRoot MTA, though their study was in vitro and may not fully replicate in vivo conditions [19]. In comparing pulpal responses, the study found mild inflammatory response was high in Group III and none in Group II. Basal Zeater *et al.* (2022) found that TheraCal LC had the lowest percentage of viable cells among the materials studied [20]. This could be due to the limited calcium release from the material due to incomplete hydration, inability to provide a proper seal due to polymerization shrinkage during setting, and presence of resin monomer components with insufficient polymerization which can cause them to accumulate to toxic levels, disrupting cellular detoxification process and leading to cell death. Leaching out of camphorquinone and ethyl-4- (dimethylamino) benzoate from TheraCal LC, can also explain its proinflammatory action [15, 16, 17, 18, 19- 20].

## Conclusion:

Data shows that all the materials tested in the study were able to induce reparative dentine formation and maintain pulp vitality. Given its novelty, conducting extensive studies on Tristrontium Aluminate, with a larger sample size, longer follow-up periods, with quantitative analysis of the dentinal bridge formation will offer valuable insights into its efficacy and potential applications in the future.

Authors' contributions: Prerna Goel and Navneet Kukreja concepts, design, and definition of the intellectual content of the study; Sushruth Nayak, Surinder Sachdeva, and Urvashi Sukhija contributed to literature search, clinical studies, experimental studies of the study and performed the study; Navneet Kukreja, Prerna Goel and Swati Chhabra data acquisition, data analysis, statistical analysis, manuscript preparation and manuscript editing. All authors critically revised the manuscript, approved the final version to be published, and agreed to be accountable for all aspects of the work. Prerna Goel and Swati Chhabra take responsibility for the integrity of the work as a whole from inception to published article and are designated as 'guarantors'.

## Figures and Tables

**Table 1:** CBCT, pulpal response, dentinal bridge formation in group I (Biodentine), Group II (MTA), Group III (TheraCalLC) and Group IV (Tristrontium aluminate)

		**n**	**Mean**	Std. Deviation*	**Minimum**	**Maximum**	**P Value**
	Group I (Biodentine)	10	1.8	0.4216	1	2	
CBCT SCORE	Group II (MTA)	10	1.7	0.483	1	2	
	Group III (TheraCalLC)	10	1.4	0.5164	1	2	
	Group IV (Tristrontium aluminate)	10	1.6	0.5164	1	2	
PULPAL RESPONSE (HISTOPATHOLOGICAL EVALUATION)	Group I (Biodentine)	10	0.1	0.3162	0	1	
	Group II (MTA)	10	0	0	0	0	0.076
	Group III (TheraCalLC)	10	0.4	0.5164	0	1	
	Group IV (Tristrontium aluminate)	10	0.1	0.3162	0	1	
DENTINAL BRIDGE (HISTOPATHOLOGICAL EVALUATION)	Group I (Biodentine)	10	1.8	0.4216	1	2	
	Group II (MTA)	10	1.8	0.4216	1	2	
	Group III (TheraCalLC)	10	1.6	0.5164	1	2	
	Group IV (Tristrontium aluminate)	10	1.8	0.4216	1	2	
*Standard deviation, p<0.05
